# A vasculogenic mimicry prognostic signature associated with immune signature in human gastric cancer

**DOI:** 10.3389/fimmu.2022.1016612

**Published:** 2022-11-23

**Authors:** Jie Wang, Wei Xia, Yujie Huang, Haoran Li, Yuchen Tang, Ye Li, Bin Yi, Zixiang Zhang, Jian Yang, Zhifei Cao, Jian Zhou

**Affiliations:** ^1^ Department of General Surgery, The First Affiliated Hospital of Soochow University, Suzhou, Jiangsu, China; ^2^ Department of Pathology, The Second Affiliated Hospital of Soochow University, Suzhou, Jiangsu, China

**Keywords:** vasculogenic mimicry, SERPINF1, TFPI2, gastric cancer, nomogram, immune infiltration

## Abstract

**Background:**

Gastric cancer (GC) is one of the most lethal malignant tumors worldwide with poor outcomes. Vascular mimicry (VM) is an alternative blood supply to tumors that is independent of endothelial cells or angiogenesis. Previous studies have shown that VM was associated with poor prognosis in patients with GC, but the underlying mechanisms and the relationship between VM and immune infiltration of GC have not been well studied.

**Methods:**

In this study, expression profiles from VM-related genes were retrieved from The Cancer Genome Atlas (TCGA) and Gene Expression Omnibus (GEO) databases. Cox regression was performed to identify key VM-related genes for survival. Subsequently, a novel risk score model in GC named VM index and a nomogram was constructed. In addition, the expression of one key VM-related gene (serpin family F member 1, SERPINF1) was validated in 33 GC tissues and 23 paracancer tissues using immunohistochemistry staining.

**Results:**

Univariate and multivariate Cox regression suggested that SERPINF1 and tissue factor pathway inhibitor 2 (TFPI2) were independent risk factors for the prognosis of patients with GC. The AUC (> 0.7) indicated the satisfactory discriminative ability of the nomogram. SsGESA and ESTIMATE showed that higher expression of SERPINF1 and TFPI2 is associated with immune infiltration of GC. Immunohistochemistry staining confirmed that the expression of SERPINF1 protein was significantly higher in GC tissues than that in paracancer tissues.

**Conclusion:**

A VM index and a nomogram were constructed and showed satisfactory predictive performance. In addition, VM was confirmed to be widely involved in immune infiltration, suggesting that VM could be a promising target in guiding immunotherapy. Taken together, we identified SERPINF1 and TFPI2 as immunologic and prognostic biomarkers related to VM in GC.

## Introduction

Gastric cancer (GC) is one of the most common gastrointestinal malignant tumors worldwide. It accounts 7.7% for of cancer-related deaths in 2020 and is recognized as the fourth leading cause of cancer-related mortality (1). Surgery accompanied by systemic chemotherapy is currently the main treatment modality for GC. Nevertheless, recurrence and metastasis are still often occurred especially in patients in the advanced stage (2, 3). Unfortunately, other treatment options such as target therapy and immunotherapy are restricted because of drug resistance (4). Therefore, it is of great necessity to understand the underlying mechanisms involved in the recurrence and resistance of GC.

Malignant solid neoplasms depend on blood and oxygen supply to maintain growth and promote metastasis. Endothelium-dependent tumor angiogenesis has long been thought to be the sole pattern of blood supply (5). However, the clinical efficacy of anti-angiogenic targeted therapy for GC is still unsatisfactory, which could be explained by that the tumor hypoxic microenvironment further exacerbates the genetic instability of gastric tumor cells and activates the tumor driver gene, causing GC to be resistant to chemotherapy as well as anti-angiogenic target therapy (6). Recent studies have revealed that highly aggressive tumor cells can form a vascular-like channel through their deformation and extracellular matrix remodeling to meet their energy demand, which is called vasculogenic mimicry (VM) (7, 8). VM, an epithelial-independent tumor microcirculation pattern, can promote tumor growth and facilitate metastasis by providing blood perfusion and promoting the secretion of protein hydrolases by tumor cells to degrade the basement membrane and extracellular matrix (9). It was found that VM is mostly present in highly malignant tumor tissues and is closely associated with tumor metastasis, recurrence and patient prognosis (10, 11). In recent studies, VM formation was reported to be closely associated with poor prognosis in tumors such as glioblastoma (12), breast cancer (13), lung cancer (14), colorectal cancer (15), gallbladder cancer (16), and so on. Various VM-related genes, including VEGF, cadherin 5 (CDH5), tissue factor pathway inhibitor 2 (TFPI2), c-myc, hypoxia-inducible factor (HIF)-1alpha, Nodal, Twist, serpin family F member 1 (SERPINF1), and mutant fibronectin ED-B, were reported to be involved in VM process (10, 11). Xu et al. reported that VEGF could induce VM formation by the PI3K signal transduction pathway (17). CDH5 is highly expressed in many aggressive cancer cells and its knockdown prevented VM (10, 11). TFPI-2 has been reported to produce some of the phenotypic changes associated with aggressive, vasculogenic melanoma cells, thus contributing to VM plasticity (18). However, to date no study has reported the synergistic effect of these VM-related genes.

This study collected VM-related genes from literature research in PubMed (18–31) and investigated VM in GC using databases including The Cancer Genome Atlas (TCGA) and the Gene Expression Omnibus (GEO). Through Cox regression, VM-related genes were selected, and based on that, a nomogram was constructed to predict the prognosis of GC. In addition, the correlation of VM-related genes with immune checkpoints and immune infiltration was evaluated. This study highlights a functional role for the VM-related gene signature and uncover a potential prognostic biomarker for GC, providing novel insights into potential therapeutic targets and strategies for the treatment of GC.

## Materials and methods

### Acquisition of VM-related genes and dataset preparation

We extracted 24 VM-related genes from the earlier reviews, then obtained the RNA-Seq data (HTSeq-FPKM), clinical data and survival data for patients with digestive system malignancy, including cholangiocarcinoma (CHOL), colon adenocarcinoma (COAD), esophageal carcinoma (ESCA), liver hepatocellular carcinoma (LIHC), pancreatic adenocarcinoma (PAAD), rectum adenocarcinoma (READ) and stomach adenocarcinoma (STAD) from The Cancer Genome Atlas (TCGA) database (http://portal.gdc.cancer.gov/). The Gene Expression Profiling Interactive Analysis (GEPIA) (http://gepia.cancer-pku.cn/) (32) is an interactive web that includes 9,736 tumors and 8,587 normal samples from TCGA and the GTEx projects. We used GEPIA to detect the outcome with VM-related genes and generate survival curves. The R package “pheatmap” was used to draw heatmaps. In addition, 431 GC samples were also obtained from the Gene Expression Omnibus (GEO, https://www.ncbi.nlm.nih.gov/geo) with the accession number of GSE84437.

### GO and KEGG enrichment analysis

Gene Ontology (GO) (33) and Kyoto Encyclopedia of Genes and Genomes (KEGG) enrichment analysis (34) were performed by the “clusterProfiler” (an R package). GO has three independent branches: molecular function (MF), biological process (BP), and cellular component (CC). The KEGG database facilitates the systematic analysis of the intracellular metabolic pathways and functions of the gene.

### Construction and validation of VM index in GC

Univariate Cox regression analysis was used to screen for VM-related genes in GC that were significantly associated with prognosis by “survival” R package. Moreover, Multivariate factors Cox regression analysis identified the VM-related genes in GC. KM-plotter (https://kmplot.com/analysis/) (35) was used to validate the prognostic effect of hub genes in GC. Multiplying the gene expression by its corresponding Cox regression coefficient and adding up was the risk scores which named VM index. To validate the effectiveness of the VM index, we detected the relationship of clinical information like T stage, N stage, tumor stage and overall survival from the TCGA database with the VM index and plotted by the “ggplot2” package in R. Based on the analysis of VM index, we divided patients into high and low-risk by the median VM index value. Then the VM index model was validated in the TCGA cohort and GEO cohort. Kaplan-Meier (KM) survival curves and risk score distribution model were plotted respectively by the “survival” R package. R packages “pheatmap” and “glmnet” were used to show the relationship between the expression of VM-related genes and VM index.

### Differentially expressed genes analysis based on VM index

To identify the putative biological pathways and functions of DEGs, the “limma” R package was performed between high and low-risk VM index groups in GC. P-value ≤0.05 was selected as the threshold value.

### Gene set enrichment analysis

The selected KEGG gene set was downloaded from the Molecular Signatures Database (MSigDB), and GSEA (version 4.0.3) (36, 37) was performed to explore the potential molecular mechanisms in the high and low VM index groups and to acquire pathways for up and down regulation. A false discovery rate (FDR) of <0.05 was considered statistically significant.

### Evaluation of microenvironment and immune cells infiltration

To evaluate the microenvironment of GC, the “ESTIMATE” R package (38) was used to acquire the tumor mutation burden (TMB), immune score, stromal score and estimate score. Then we used “single sample GESA (SsGESA)” and “pheatmap” packages to estimate differences in the infiltration of 29 immune cell types between high and low VM index groups. The Stemness index of the tissue samples containing DNA methylation-based stemness scores (DNAss) and mRNA expression-based stemness scores (RNAss) was also assessed (39). We also detected the relationship between immune checkpoints and the VM index from the data of the TCGA database.

### Development and validation of the nomogram model

We incorporated all statistically significant clinicopathological parameters identified *via* multivariate Cox analysis and further established a visualized nomogram model including T stage, N stage and VM index through “rms” and “survival” R package, thus predicting the 3-, 5-year overall survival (OS) probability of patients. The Receiver Operating Characteristic (ROC) Curve of the nomogram was plotted to estimate the nomogram’s predictive abilities with respect to GC patients’ prognosis.

### Immunohistochemical staining

Immunohistochemical (IHC) staining was performed in paraffin-embedded tissue from patients with GC at the Second Affiliated Hospital of Soochow University (Suzhou, China) between April 2017 to July 2018 to measure SERPINF1 protein level using an anti-SERPINF1 antibody (1:200, Abcepta, Suzhou, China). There were 33 cases of GC and 23 samples of paracancer tissues. All tissues were collected from patients who had not received chemotherapy or radiotherapy prior to surgery in the Second Affiliated Hospital of Soochow University and the informed consents were signed by all patients. Detailed clinicopathologic characteristics of the patients were listed in **Supplementary Table 1**. Each GC sample was evaluated based on the staining intensity and the percentage of cells with. The H-score was calculated as previously reported (40). The H-score value ranges between 0 and 300. Unpaired t-test was used to compare the SERPINF1 expression in GC tissues and the paracancer tissues.

### Statistical analysis

Statistical analyses were carried out using R software (version 4.0.5; http://www.Rproject.org). Correlation with survival was evaluated by means of Kaplan–Meier plots, log-rank test, and univariate and multivariate analyses based on the Cox proportional hazards method. Student’s t test was applied for categorical variables. The correlation between two variables was assessed using Spearman’s correlation test. In this study, P < 0.05 was identified as statistically significant.

## Results

### The expression level of VM-related genes and functional enrichment analysis

**Figure 1:**
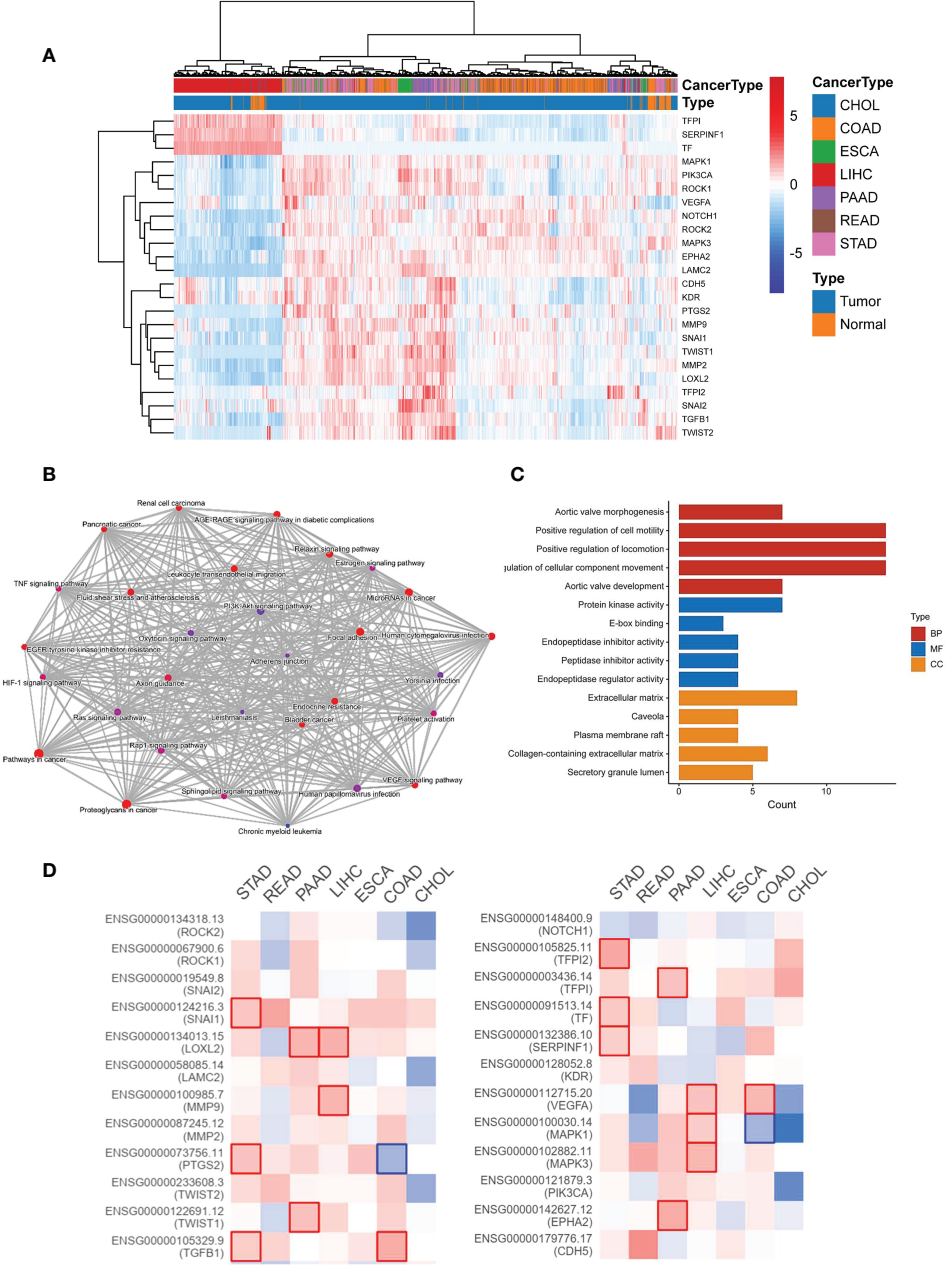
The expression and function of 24 VM-related genes in digestive system malignancies. **(A)** Heatmap showing the clustering in digestive system malignancy based on the expression of 24 VM-related genes. **(B)** Network of KEGG enriched terms colored according to clusters. **(C)** Gene Ontology (GO) analysis including BP, CC, and MF. BP, biological process; CC, cellular component; MF, molecular function. **(D)** Expression level of VM-related genes and outcomes in GEPIA.

To detect the expression level of VM-related genes in digestive system cancers and normal tissues, we draw a heatmap by the data from TCGA and found out that VM genes were highly expressed in varied digestive system cancers, including CHOL, COAD, ESCA, LIHC, PAAD, READ, STAD and so on (**Figure 1A**). Enrichment analyses including GO and KEGG was performed to investigate the functions and involved pathways of VM-related genes and their potential relationship with GC development. KEGG pathways analysis was carried out to investigate comprehending functions of VM-related genes and the results showed that VM genes were involved in TNF signaling pathways, HIF-1 signaling pathway, VEGF signaling pathway, and focal adhesion (**Figure 1B**). GO analysis revealed that the top five enriched BP terms were “aortic valve morphogenesis”, “positive regulation of cell motility”, “positive regulation of locomotion”, “regulation of cellular component movement” and “aortic valve development”. The top five enriched MF terms were “protein kinase activity”, “E-box binding”, “endopeptidase inhibitor activity”, “peptidase inhibitor activity” and “endopeptidase regulator activity”. The top five enriched CC terms were “extracellular matrix”, “caveola”, “plasma membrane raft”, “collagen-containing extracellular matrix” and “secretory granule lumen” (**Figure 1C**). Additionally, we found out that high expression levels of VM-related genes bring worse outcomes through GEPIA analysis (**Figure 1D**).

### Identification of VM-related hub genes in GC

**Figure 2:**
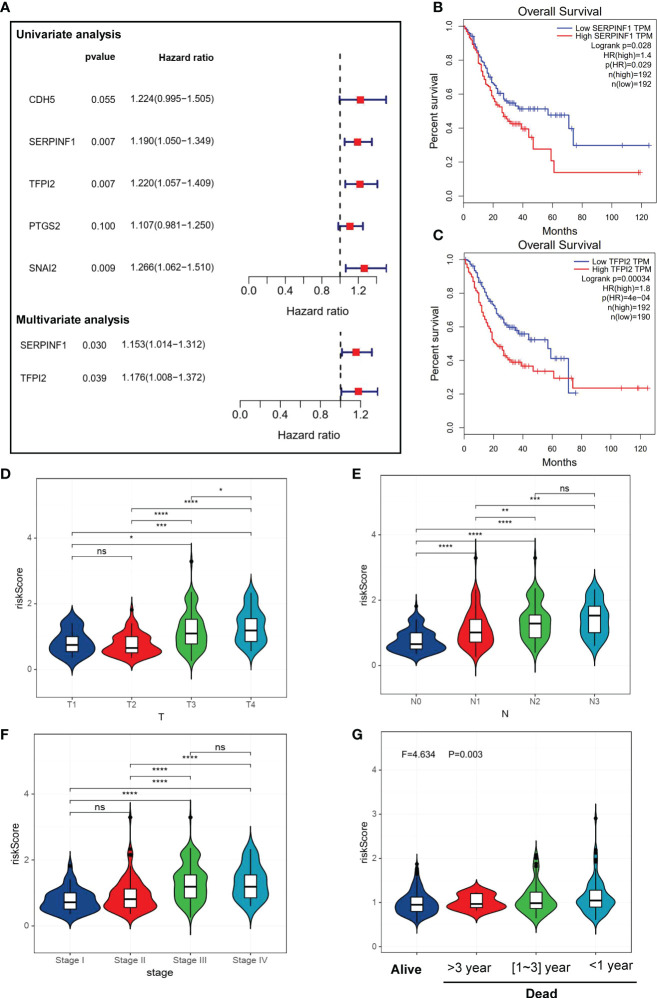
Identification of VM-related genes in GC. **(A)** A forest map of the most important VM-related genes found by Univariate and Multivariate Cox regression analysis in GC. **(B, C)** Kaplan-Meier survival analysis showed that high expression level of SERPINF1 and TPFI2 were correlate to worse overall survival. **(D–G)** Violin plots of VM-index with clinical factors. **(D)** stage of GC. **(G)** VM-index with survival status in GC. VM-index with T stage of tumor. **(E)** VM-index with N stage of tumor. **(F)** VM-index with tumor stage. **(G)** VM-index with survival status. * means P < 0.05, ** means P < 0.01, *** means P < 0.001, **** means P<0.0001. ns indicates not significant (p>0.05).

Our univariate Cox regression analysis showed that 5 VM-related genes were correlated with the prognosis of patients with GC (**Figure 2A**), including SERPINF1, TFPI2, CDH5, prostaglandin-endoperoxide synthase 2 (PTGS2) and snail family transcriptional repressor 2 (SNAI2). Furthermore, we conducted the multivariate Cox regression on the genes obtained in the previous steps, and we retained SERPINF1 and TFPI2 as the hub genes of VM in GC (**Figure 2A**). We also detected the relationship between expression levels of these two VM-related genes with OS status (**Figures 2B, C**). Also, expression levels of SERPINF1 and TFPI2 were associated with worse OS and progression-free survival (PFS) (**Figures S1A–D**). These results suggested that SERPINF1 and TFPI2 are risk factors in GC.

### Construction and validation of a risk prediction model named VM index

**Figure 3:**
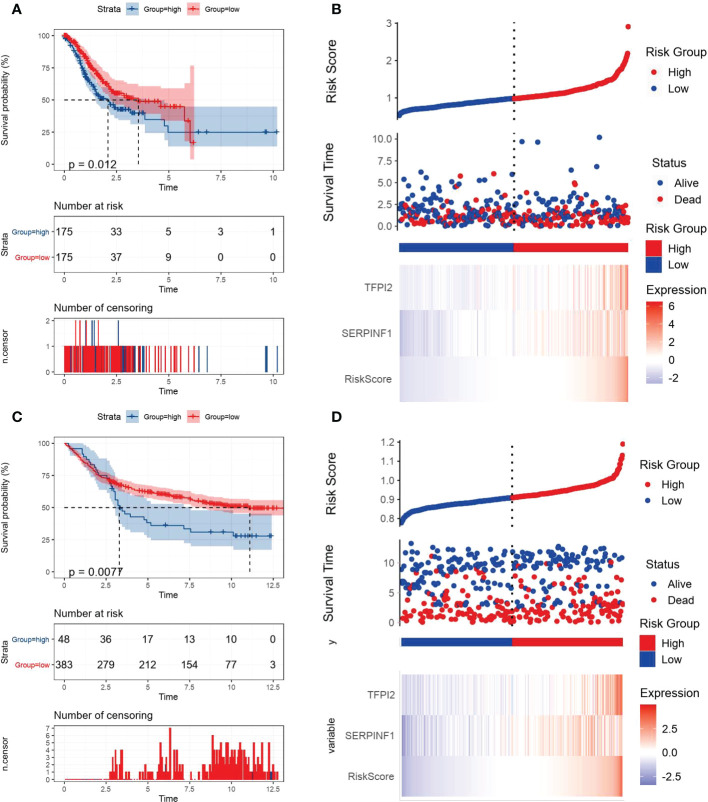
Kaplan-Meier plots and risk assessment model for prognosis prediction in the TCGA and GEO databases. All GC cases were stratified in to high- and low-VM index groups based on median risk score in **(A, B)** the TCGA training cohort and **(B, C)** the GEO testing cohort. **(A, C)** Kaplan-Meier curves revealed that individuals with high risk displayed diminished survival probability than those with low VM index. **(B, D)** Risk survival status plots were consistent with the heatmaps for genes expression.

VM index was constructed based on the two VM hub genes (SERPINF1 and TFPI2), which means VM index is the sum of proportional expression of SERPINF1 and TFPI2. We found that higher T stage, N stage, Tumor stage and poorer overall survival promoted the increase of VM index (**Figures 2D–G**), which indicated that higher VM index represented poor prognosis, revealing the potential of VM index in prognosis prediction. To verify the effectiveness of the VM index prognosis prediction, Kaplan-Meier curves for the training cohorts in TCGA and validation cohorts in the GEO database were performed and the results were shown in **Figures 3A–C**. VM index was negatively correlated with prognosis. We then divided patients into high and low-risk groups according to the best cut-off value of the VM index. The distribution of the survival data and VM index for each patient, as well as the heatmaps of SERPINF1 and TFPI2, are shown in **Figures 3B–D**, in which patients with higher VM index usually had shorter survival time. These results verified that SERPINF1 and TFPI2 are important risk factors of GC.

### Correlations of VM index with DEGs in GC and tumor microenvironment

**Figure 4:**
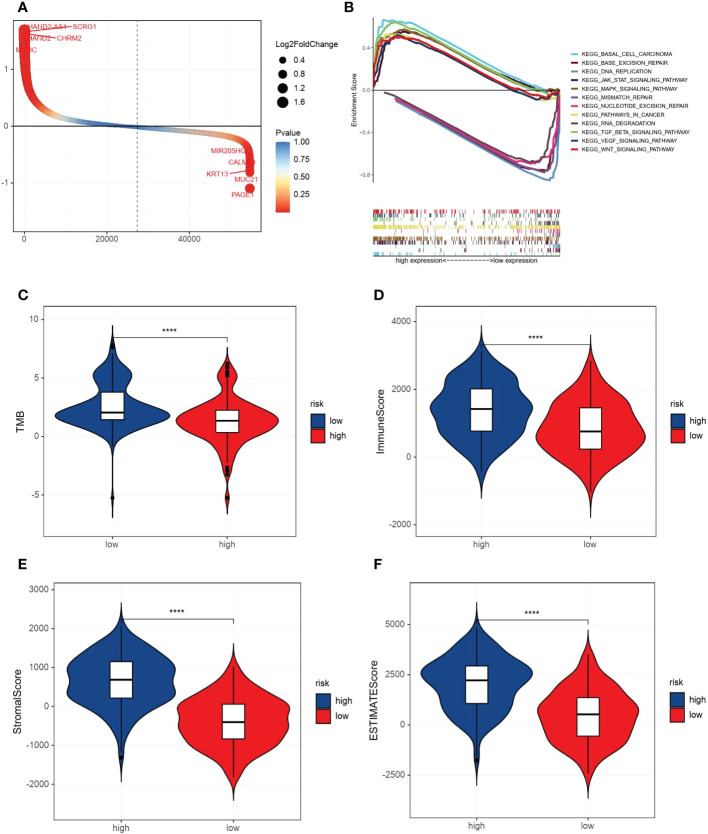
Analysis of DEGs, GESA and correlation with TMB and immune/stromal scores. **(A)** DEGs according to VM index. **(B)** Gene set enrichment analysis (GSEA) results of the difference enrichment pathways according to VM index. **(C)** Distribution of TMB according to VM index. **(D)** Distribution of immune score according to VM index. **(E)** Distribution of stromal score according to VM index. **(F)** Distribution of ESTIMATE score according to VM index. **** means P<0.0001.

By comparing the gene expression levels of high and low VM index groups, we screened DEGs and the most five highly expressed genes were HAND2 antisense RNA 1 (HAND2-AS1), stimulator of chondrogenesis 1 (SCRG1), heart and neural crest derivatives expressed 2 (HAND2), cholinergic receptor muscarinic 2 (CHRM2) and myocilin (MYOC) (**Figure 4A**). To further explore the difference in enrichment pathways between high and low VM index groups, GESA analysis was performed. In the high VM index group, pathways mainly related to tumor metastasis and angiogenesis included basal cell carcinoma, TGF-β-signaling pathway, MAPK-signaling pathway, VEGF-signaling pathway, pathways in cancer, WNT-signaling pathway and JAK-STAT-signaling pathway. In contrast, the low VM index group was mainly related to gene repair such as base excision repair, nucleotide excision repair, RNA degradation, mismatch repairs and DNA replication (**Figure 4B**).

Then, ESTIMATE was performed to compare TMB, immune and stromal scores in two groups. As shown in the diagrams, a high VM index was negatively associated with TMB, while positively correlated with the immune score, stomal score and ESTIMATE score (**Figures 4C–F**). Also, SsGESA and ESIMATE were conducted to examine the relationship between the expression of SERPINF1 and TFPI2 with tumor microenvironment in GC. Higher expression of SERPINF1 and TFPI2 was accompanied by lower cancer stemness while with a higher stromal score, immune score and ESTIMATE score (**Figure S2**).

### VM index and immune cell infiltration analysis

**Figure 5:**
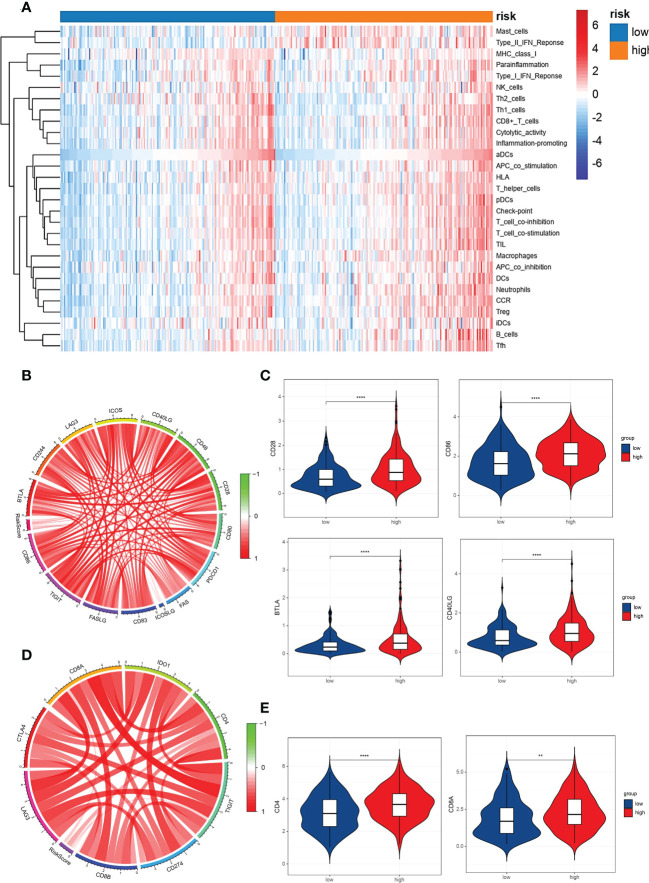
Correlation analysis between the VM index, immune cells infiltration in GC and pivotal immunotherapy-associated molecules. **(A)** Heatmap for immune cell infiltration in GC clustered by VM index. Red color represents positive correlation and blue color represents negative correlation. Relationship between VM index expression and immune cell infiltration. **(B, D)** Circular plots showed that VM index was correlated with immunotherapy-associated molecules in TCGA database; **(C, E)** Higher VM index was correlated with higher expression of immune checkpoints in GC, including CD28, CD86, BTLA, CD40LG, CD4 and CD8A. ** means P < 0.01, **** means P<0.0001.

For further understanding of the relationships between immune cell infiltration and VM index, data from TCGA was used to quantify the activity or enrichment levels of immune cells, functions and pathways in GC by R package SsGESA. Heatmap and box plot of 29 kinds of immune cells indicated that the VM index was correlated to immune response (**Figure 5A**). We further examined the relationships of immune cells and checkpoints with the VM index, and the results showed that the expression of immune checkpoints including CD28, CD86, BTLA, CD40LG, CD4 and CD8A was positively correlated with the VM index (**Figures 5B–E**).

### Construction of a nomogram for GC

**Figure 6:**
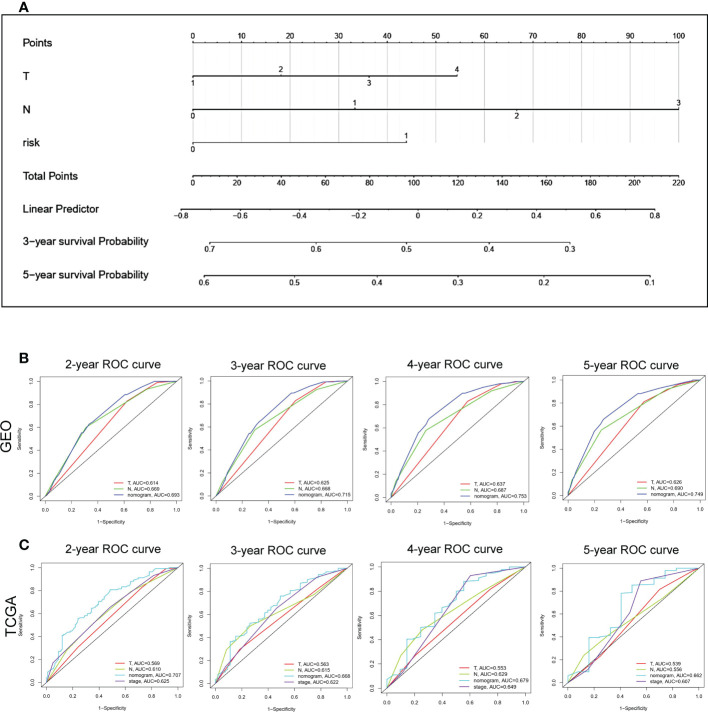
Construction of a nomogram based VM-index for prognosis prediction of GC. **(A)** The nomogram using T stage, N stage and VM index. For each patient, three lines are drawn upward to verify the points received from the three predictors of the nomogram. The sum of these points situates on the ‘Total Points’ axis. Then a line is drawn downward to assess the 3-, and 5-year overall survival of GC. **(B, C)** The ROC curve to evaluate the nomogram in GEO and TCGA database. Y-axis, Sensitivity; X-axis, Specificity.

To quantify the risk assessment and survival status for GC patients, a nomogram was built with VM index (**Figure S3**) as well as other clinicopathological features including T stage, and N stage (**Figure 6A**). In the nomogram model, the above parameters were assigned scores, and the score of each parameter was based upon plotting upward a straight line. For each GC patient, the survival probability of 3 and 5 years was estimated through the drop line from the total points line to the result line. To prove the accuracy of the nomogram, we made a comparison of the ROC curves between our nomogram model and additional clinicopathological variables (T stage, N stage and tumor stage) in GEO (**Figure 6B**) and TCGA (**Figure 6C**) cohorts. With the area under the curve (AUC) of the nomogram (combined model) larger than any single factor, our nomogram model, constituted by VM index, T stage, and N stage, was an optimal model to predict the long-term OS of GC patients.

### Validation of the expression of SERPINF1 in GC tissues

**Figure 7:**
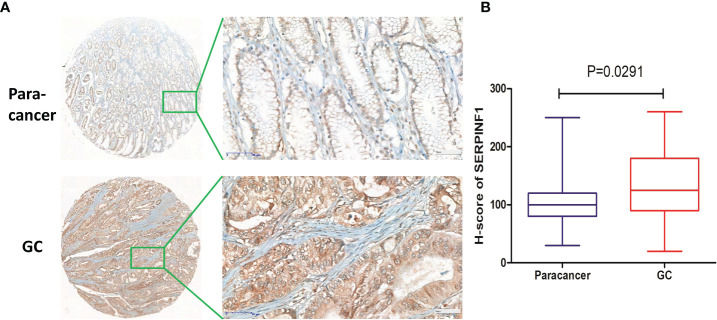
Immunohistochemical staining of SERPINF1 in GC tissues and paracancer tissues. **(A)** The immunohistochemical staining results of SERPINF1 in GC and paracancer tissues. **(B)** Statistical significance was determined by unpaired t-test. Data are expressed as means ± SD.

To further identify the VM-related hub genes in the pathologenesis of GC, the expression of one hub gene (SERPINF1) was assessed in 33 GC tissues and 23 paracancer tissues using an IHC staining assay. Our IHC staining results on the GC tissue microarray demonstrated that SERPINF1 expression in GC tissues was obviously higher than that in adjacent non-tumor tissues (P<0.05, **Figures 7A, B**). Generally, the data indicated that SERPINF1 could be the candidate biomarkers for the VM process of GC.

## Discussion

GC is one of the most common malignancies and acts as the leading cause of cancer-related death worldwide (1). Although surgery accompanied by systemic chemotherapy is recommended as the main treatment method for GC currently (4, 41), recurrence and metastasis still happen in patients with resected GC and remains disturbing. Target therapies and immunotherapies have been applied as standard treatment for systemic therapy for unresectable locally advanced, recurrent or metastatic GC (1, 42–44), but unfortunately, the clinical efficacy of anti-angiogenic therapy is unsatisfactory due to drug resistance caused by hypoxic tumor microenvironment (45–47). Therefore, the identification of novel factors for prognosis prediction of patients with GC is of great necessity.

VM, an alternative blood supply to tumors that is independent of endothelial cells or angiogenesis, has been demonstrated as a critical factor involved in the pathogenesis of solid tumors and is significantly associated with increased resistance to chemotherapy, low survival, and poor prognosis of patients with malignant tumors (7, 48, 49). It was reported that VM could promote tumor neovascularization to favor metastasis (50) and to drive resistance to antiangiogenic therapy (12, 51). A meta-analysis showed that VM was related to the poor OS and DFS of patients with digestive cancer (52). Baeten et al. reported that VM formation could be used to predict prognosis in colorectal cancer (15). To date, only one review has reported that VM is associated with a poor prognosis in patients with GC in China (53). The underlying mechanisms and the relationship between VM and immune infiltration is still unclear. In this study, we demonstrated that VM-related genes as previously reported were upregulated in GC and involved in the HIF-1 signaling pathway (54) and VEGF signaling pathways (55). Two VM-related genes, SERPINF1 and TFPI2, were identified as independent risk factors for the prognosis of patients with GC through Cox regression analysis (18, 56). SERPINF1 was reported to be involved in the migration and invasion by extracellular matrix (ECM) remodeling in GC (19). TFPI2 may take part in promoting colorectal cancer by changing the DNA methylation status of colon epithelial cells (39, 57, 58). In our study, we constructed a model named VM index which could well evaluate the expression levels of VM in GC. Interestingly, for the first time, we found that the VM index was correlated with immune cells and immune checkpoints such as CD28, CD86, BTLA, CD40LG, CD4, and CD8A in GC, suggesting that VM may promote the pathogenesis and metastasis of GC through regulating immune cells and immune surveillance. We also demonstrated that the upregulation of VM is accompanied with lower cancer stemness indices including RNAss and DNAss, while with higher immune infiltration, including stromal score, immune score and ESTIMATE score, indicating that cancer stem cells and immune infiltration were of great necessity in the regulation of GC cells by VM.

Tumor hypoxic microenvironment, as an indispensable part of cancer that has been exclusively focused on and increasingly acknowledged for decades, was reported to be inseparable from VM (59). Herein our study analyzed the relationship between VM, several immune infiltration criteria, and the tumor microenvironment. ESTIMATE and GSEA revealed that a high VM index was negatively associated with TMB, while positively correlated with the immune score, stomal score and ESTIMATE score. Recently, a review reported that cancer stem cells have been identified to be involved in VM in gastrointestinal cancer (45). Consistent with that, our study revealed the correlation between the two VM key genes (SERPINF1 and TFPI2) and stemness. Besides, the VM index was identified to be positively associated with an immune score, stromal score and ESTIMATE score, which are important metrics of the tumor microenvironment (38). VM, an alternative mechanism of vasculatures, has been reported to be involved in resistance to anti-angiogenic therapies, whereas the combination of anti-angiogenesis and immune therapy could bring out better clinical efficacy (60–63). Our study further investigated the regulatory role of VM in immune cells. There are several possible mechanisms by which the VM index is involved in immune infiltration. On the other hand, the VM index could upregulate immune checkpoints including CD28, CD86, BLTA and CD40LG to inhibit immune response, thus leading to tumor immune escape. The VM index could also directly upregulate immunotherapeutic genes including CD4 and CD8A. Both of these two mechanisms provide potential targets and novel insights into the treatment of VM by immunotherapy.

To explore the impact of VM on the prognosis of patients with GC, a nomogram based on VM index, T stage and N stage was constructed to visualize the effects of clinical features and VM index on patients’ 3- and 5-year survival probabilities. Time-dependent AUC identified that our nomogram had high prediction efficiency and was better than the T stage and N stage, demonstrating the potential value of our nomogram in clinical practice. Furthermore, this study has allowed the development of strategies with therapeutic potential directed against VM formation. In clinic, the combination of traditional anti-angiogenic therapies with SERPINF1 and TFPI2, the two potential anti-VM targets may improve the outcomes of patients with GC. Besides, our nomogram may be a valuable tool for assessing the prognosis of patients with GC to date, and researchers and clinicians may conveniently access it.

## Conclusion

In summary, our study constructed a novel risk score model in GC named VM index based on SERPINF1 and TFPI2. The VM index showed satisfactory predictive performance in both training and validation cohorts and could be applied to predict the prognosis and tumor microenvironment in patients with GC. Moreover, a precise and convenient nomogram based on VM index and clinical factors including the T stage and N stage was well constructed and validated by the ROC curve, in which the nomogram showed excellent performance in the prediction of GC compared with merely the T stage or N stage. In addition, we confirmed that VM is widely involved in the regulation of immune infiltration and immune checkpoints. Therefore, we have not only constructed a precise and convenient model for prognosis prediction but also have proposed that VM could be a promising molecular target in guiding immunotherapy.

## Data availability statement

Publicly available datasets were analyzed in this study. This data can be found here: TCGA (http://portal.gdc.cancer.gov/) and Gene Expression Omnibus (https://www.ncbi.nlm.nih.gov/geo) under the accession number GSE84437.

## Ethics statement

The studies involving human participants were reviewed and approved by the Second Affiliated Hospital of Soochow University. The patients/participants provided their written informed consent to participate in this study.

## Author contributions

Conceptualization: JZ, ZC, and JW; methodology: JZ, JW and WX; data acquisition material support: JW, WX, YH, HL, YT, YL and BY; data analyses and interpretation: JW, YH and YT, writing original draft preparation: JW; writing – review & editing: JZ, ZC and ZZ; supervision: JZ; funding acquisition: JZ and ZC. All authors contributed to the article and approved the submitted version.

## Funding

This study was supported by the Project of the National Natural Science Foundation of China (grant number: 81902385); the Foundation Research Project of the Natural Science Foundation of Jiangsu Province (grant number: BK20201173); and the Project of Medical Research of Jiangsu Province (grant number: Y2018094).

## Conflict of interest

The authors declare that the research was conducted in the absence of any commercial or financial relationships that could be construed as a potential conflict of interest.

## Publisher’s note

All claims expressed in this article are solely those of the authors and do not necessarily represent those of their affiliated organizations, or those of the publisher, the editors and the reviewers. Any product that may be evaluated in this article, or claim that may be made by its manufacturer, is not guaranteed or endorsed by the publisher.
