# Supplementary material for: A vasculogenic mimicry prognostic signature associated with immune signature in human gastric cancer
Source: Front Immunol. 2022 Nov 23;13:1016612. doi: 10.3389/fimmu.2022.1016612 (PMC9727221; doi:10.3389/fimmu.2022.1016612)
Supplement: Supplementary file 1 [file DataSheet_1.pdf]

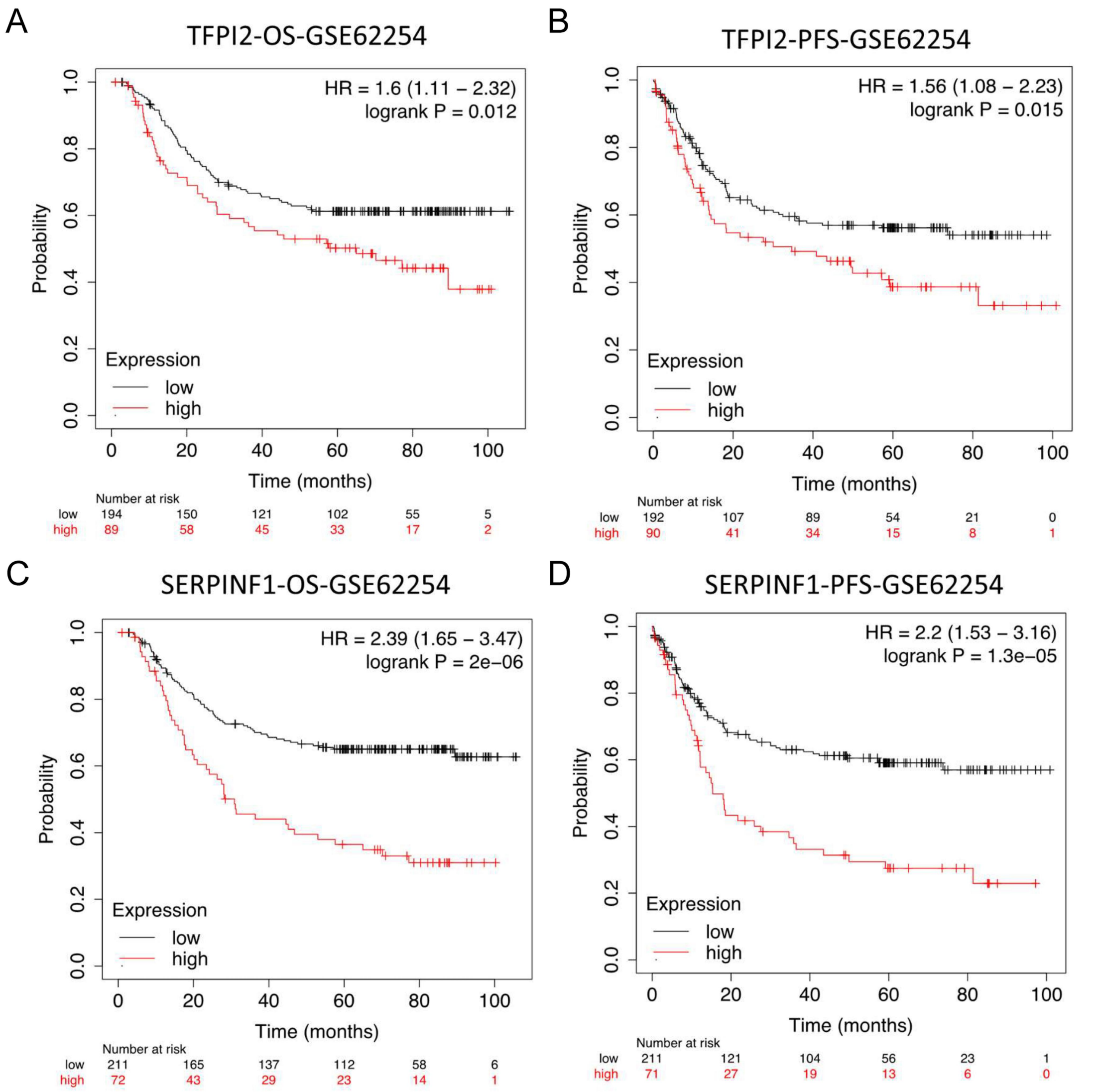

Supplementary Figure 1 Correlation of expression level of SERPINF1 and TFPI2 with Overall Survival (OS) and Progression-Free-Survival (PFS) in GC.

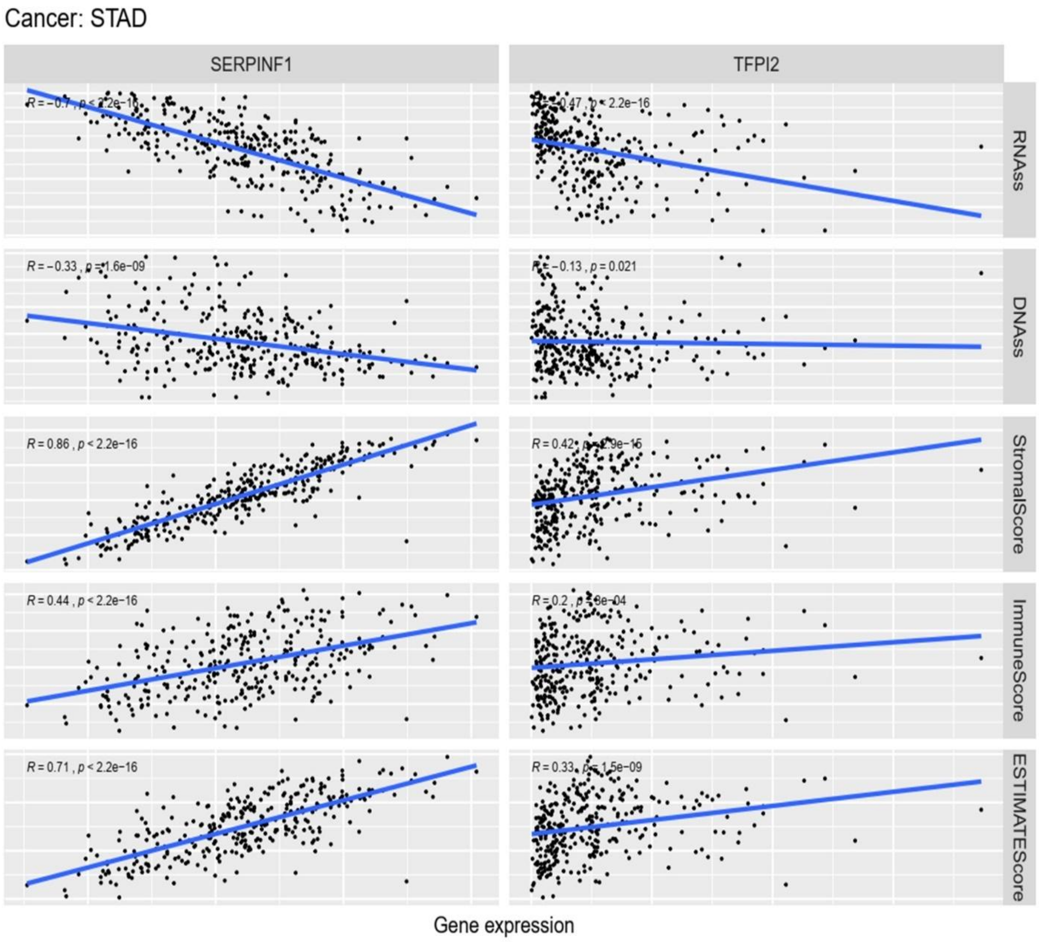

Supplementary Figure 2 Stemness and microenvironment with SERPINF1 and TFPI2 in GC.

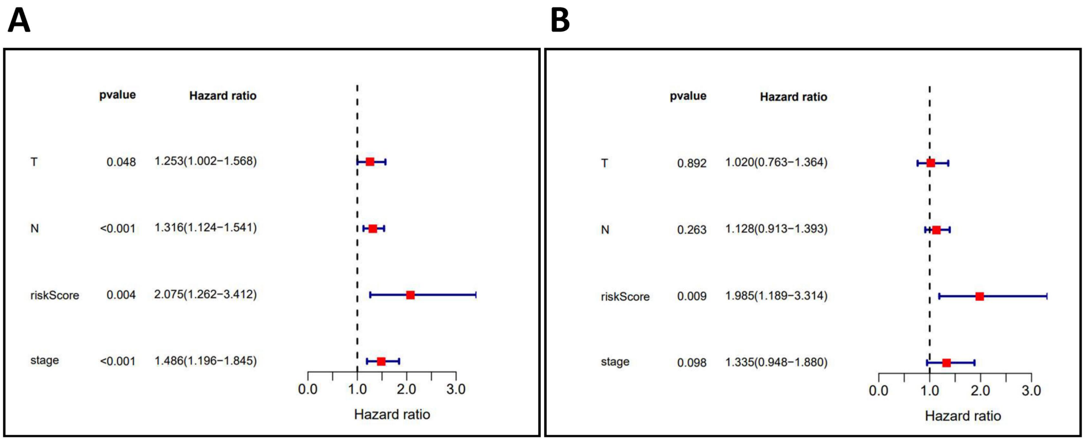

Supplementary Figure 3 Univariate (A) and multivariate (B) analysis showed that VM index is an independent prognostic indictor for GC patients.

Supplementary Table 1 Clinicopathologic characteristics of the GC patients.

**Clinicopathologic parameters**		**n**
All cases		33
Age (year)	<60	18
	》60	15
Gender	Male	24
	Female	9
Depth of invasion	T1+T2	10
	T3+T4	23
TNM staging	I+II	13
	III+IV	20
Lymph node metastasis	Negative	12
	Positive	21
